# Surgical Nuances in Ultrasound-Guided Percutaneous Distal Catheter Placement in Pediatric Ventriculoatrial Shunts

**DOI:** 10.7759/cureus.84345

**Published:** 2025-05-18

**Authors:** Lagree G Reynoso, Ariadna Rodríguez Lezama, Carlos Andres Hernández Martínez, Emmely Alexandra Prado, Mauricio Matus, Edgard Herrera

**Affiliations:** 1 Neurosurgery, Hospital Militar Escuela Dr. Alejandro Dávila Bolaños, Managua, NIC

**Keywords:** fluoroscopy guided, pediatric hydrocephalus, seldinger technique, ventriculoatrial shunt, ventriculoperitoneal shunt placement

## Abstract

Currently, there is no universally accepted consensus regarding the optimal site for distal catheter placement in patients with congenital hydrocephalus and a non-functional peritoneal cavity, as therapeutic strategies must be meticulously individualized based on each patient’s unique anatomical and physiological considerations. We report a complex case involving a one-year-old male infant, born prematurely, with alobar holoprosencephaly (HPE) and congenital hydrocephalus, who experienced multiple ventriculoperitoneal shunt (VPS) failures. These complications were attributed to impaired peritoneal absorption of cerebrospinal fluid (CSF), recurrent shunt infections, and occlusion of an external ventricular drainage system. As a salvage intervention, a ventriculoatrial shunt (VAS) was successfully established using ultrasound-guided internal jugular vein cannulation, followed by fluoroscopy-assisted endovascular placement of a modified distal catheter via the Seldinger technique. This approach underscores the utility of image-guided VAS as a viable alternative in cases of VPS failure secondary to peritoneal CSF resorption insufficiency, particularly in patients with complex neuroanatomical profiles.

## Introduction

Hydrocephalus remains a significant contributor to morbidity and mortality in the pediatric population, posing substantial clinical challenges [1]. Ventriculoperitoneal shunting remains the gold standard for cerebrospinal fluid (CSF) diversion in hydrocephalus management [1]. However, in certain clinical scenarios - such as congenital gastrointestinal anomalies, necrotizing enterocolitis, ascites, abdominal infections, or extensive abdominal adhesions - the peritoneal cavity may become unsuitable for distal catheter placement, thereby complicating ventriculoperitoneal shunt (VPS) procedures [2]. Moreover, additional factors, including prematurity, low birth weight, and impaired wound healing, further contribute to diminished shunt longevity and function [3,4].

In these complex cases, where traditional VPS placement is not feasible, ventriculoatrial shunting provides a viable and effective alternative. A comprehensive review of current literature reveals that, while numerous studies have explored ultrasound [5-7] and fluoroscopy [8,9] guided percutaneous ventriculoatrial shunt (VAS) placement in pediatric patients as a secondary solution, there remains a notable paucity of evidence addressing the interchangeability of catheters initially designed for femoral vein access when applied via the internal jugular vein (IJV), particularly in cases involving intricate anatomical anomalies.

This report highlights the surgical nuances involved in selecting appropriate catheters tailored to patient-specific anatomical characteristics, utilizing intraoperative ultrasound and fluoroscopic guidance. It emphasizes the importance of these techniques in ensuring precise distal catheter placement while minimizing post-surgical morbidity and optimizing long-term shunt function.

## Case presentation

A one-year-old male infant, born prematurely at 35 weeks of gestation via cesarean section, had a birth weight of 3,280 g and an Apgar score of 8/9. At birth, his measurements included a head circumference of 48 cm, a chest circumference of 29 cm, and an abdominal circumference of 28 cm. The newborn was admitted to the neonatal intensive care unit in critical condition with suspected neonatal respiratory distress syndrome.

**Figure 1:**
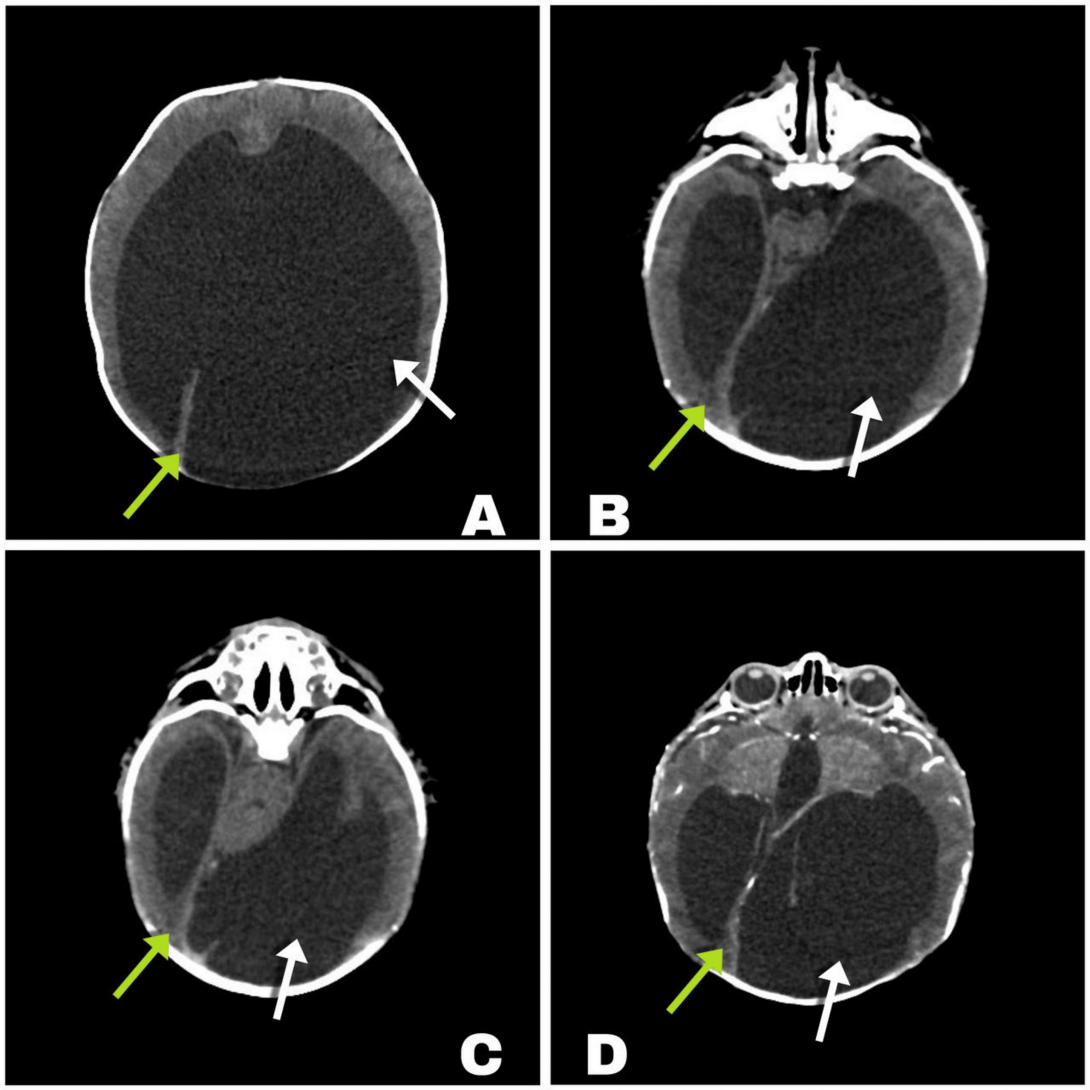
Computed axial tomography scan of the brain A-D: Alobar holoprosencephaly observed by the minimal cortical mantle. Severe supratentorial hydrocephalus with a minimal cortical mantle. White arrows indicate asymmetry on the left side. Green arrows point to the falx cerebri in various axial slices.

**Figure 2:**
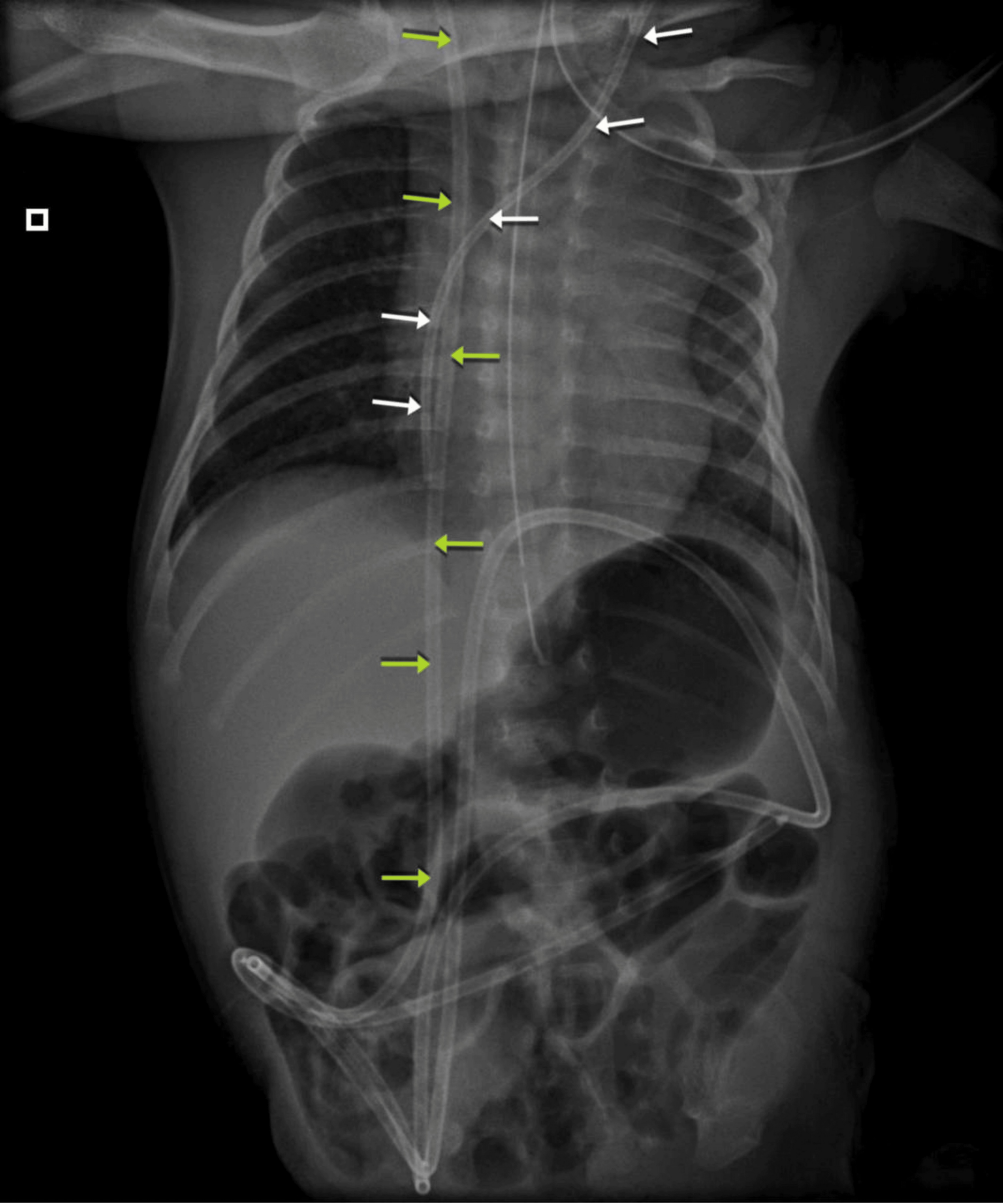
Anteroposterior chest and abdominal X-ray White arrows indicate the path of the central venous catheter. The thoracic and abdominal course of the ventriculoperitoneal shunt is also visible, marked by the green arrows.

A cranial computed tomography (CT) scan (Figure 1) revealed significant findings, including holoprosencephaly (HPE) and severe supratentorial hydrocephalus, with an estimated CSF volume of 900 mL. Despite initially opting for a conservative approach to minimize perioperative morbidity and mortality, the patient’s condition deteriorated on day 9 of life. This deterioration was marked by a sudden increase in head circumference (to 50.5 cm), along with irritability, crying, and vomiting - clinical signs consistent with intracranial hypertension. Consequently, a VPS was placed to manage the hydrocephalus, and a central venous catheter was also inserted during the procedure. The positions of both devices were confirmed via X-ray (Figure 2).

**Figure 3:**
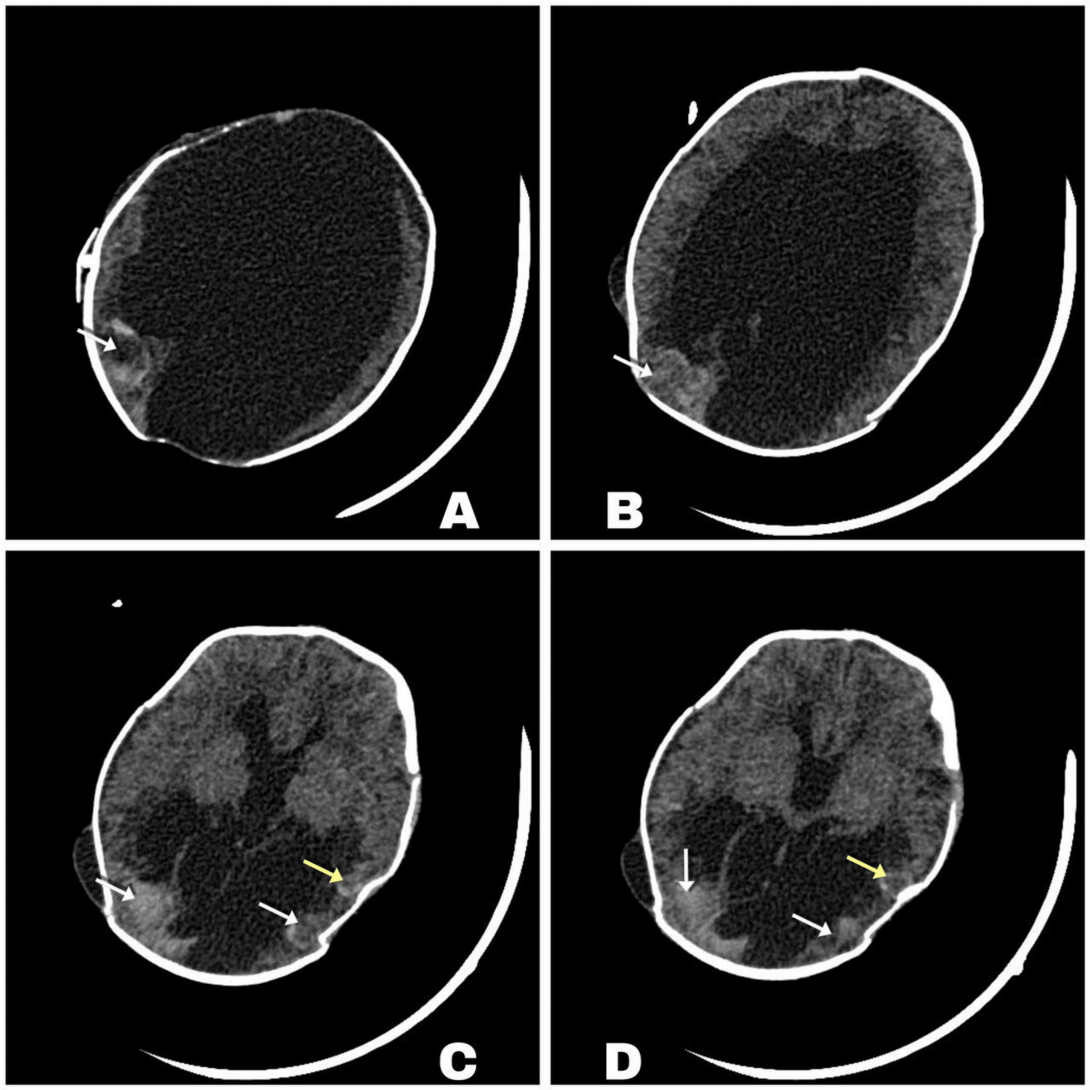
Follow-up CT scan of the brain A-D: White arrows indicate right occipito-parietal intraparenchymal hematomas and yellow arrows indicate left temporo-parietal intraparenchymal hematomas. CT: computed tomography

Follow-up imaging, including a CT scan, identified multiple foci of intraparenchymal hemorrhage (Figure 3). The patient subsequently underwent VPS revision in the operating room. During the procedure, a contained subgaleal CSF fistula was discovered and successfully repaired. Despite this intervention, the patient developed signs of acute hydrocephalus, including CSF leakage through the surgical wound and an increase in head circumference. A second VPS revision was performed, and the shunt was replaced due to suspected obstruction.

After a second VPS revision (third overall procedure), the patient’s symptoms of increased intracranial pressure (ICP) temporarily resolved. However, these symptoms reappeared with an increase in head circumference and abdominal distension. It was determined that the peritoneum, due to its immaturity, was insufficient for adequate CSF reabsorption. As a result, the VPS was removed and replaced with an external ventricular shunt (EVS) system. This new system was maintained for several weeks, allowing the patient to gain weight.

Several weeks later, xanthochromic CSF leakage was observed from the drainage system. A CSF sample was obtained, and microbiological testing revealed the growth of *Staphylococcus haemolyticus*, with leukocytosis, elevated acute phase reactants, and evidence of device-associated ventriculitis (Table 1). Antibiotic treatment with glycopeptides was initiated.

**Table 1:** Laboratory tests performed with results and reference parameters MCV: mean corpuscular volume; MCH: mean corpuscular hemoglobin; MCHC: mean corpuscular hemoglobin concentration; RDW-SD: red cell distribution width standard deviation; RDW-CV: red cell distribution width coefficient of variation; MPV: mean platelet volume; ECLIA: electrochemiluminescence immunoassay; CSF: cerebrospinal fluid

Evaluation parameters	Patient values	Normal values
Complete blood count		
Hematocrit	33.5%	39-50%
Hemoglobin	12.1 g/dL	13-17 g/dL
White blood cell count	20.53 x 10e3/uL	5-10 x 10e3/uL
Red blood cell count	4.48 x 10e6/µL	4-6.3 x 10e6/µL
VCM	74.8 fL	72-96 fL
HCM	27 pg	27-32 pg
CHCM	36.1 g/dL	32-37 g/dL
Platelets	624 x 10e3/uL	150-500 x 10e3/uL
RDW-SD	42.0%	37.2-54%
RDW-CV	15.8%	11-16%
MPV	9.4%	9-13%
Neutrophils	32.9%	55-65%
Lymphocytes	56.9%	25-35%
Monocytes	9.6%	3-10%
Eosinophils	0.4%	0.5-4%
Basophils	0.2%	0-2%
Neutrophils (count)	6 x 10e3/uL	5.5-6.5
Lymphocyte (count)	11 x 10e3/uL	20-45 x 10e3/uL
Monocyte (count)	1 x 10e3/uL	0-8 x 10e3/uL
Eosinophils (count)	0 x 10e3/uL	3-5 x 10e3/uL
Basophils (count)	0 x 10e3/uL	0-2.5 x 10e3/uL
Blood Chemistry		
C-reactive protein	0.91 mg/dL	0-0.5 mg/dL
Immunology		
Procalcitonin ECLIA	1.14 ng/mL	0.5
		< 0.5 Low risk of sepsis
		> 0.5 High risk of sepsis
CSF study		
Physical examination		
Color	Xanthochromic	Clear
Aspect	Clear	Clear
pH	8	7.4
Special chemistry		
Glucose (CSF)	31.9 mg/dL	Newborns: 60-80 mg/dL
		Adults: 40-70 mg/dL
Proteins in CSF	55.7 mg/dL	Adults: 15-45 mg/dL
		Newborns: 15-100 mg/dL
		3 months to 60 years: 15-45 mg/dL
		Over 60 years old: 15-60 mg/dl
Albumin in CSF	36.7 mg/dL	3 months to 4 years: < 45 mg/dl
		Older than 4 years: 10 to 48 mg/dl
Cell count		
Leukocytes	2 x mm^3^	Newborns 0-30 x mm^3^
		Adults 0-5 x mm^3^
Erythrocytes	1,000 x mm^3^	No red blood cells
Differential		
Polymorphonuclear	0%	Newborns: 0-8%
		Adults: 0-6%
Mononuclear	100%	Newborns: 5-35%
		Adults: 40-80%
Microscopic examination		
Crenated erythrocytes	67%	Newborns: 50-90%
		Adults: 15-45%
Bacteria	No bacteria were observed	No microorganism

Once the infection was resolved, the EVS system was revised four additional times due to repeated failures and signs of increasing ICP. Eventually, a decision was made to place a VPS, but later that day, it was replaced with an EVS due to peritoneal obstruction (Table 2). Despite these efforts, the patient continued to experience complications, leading to repeated EVS replacements and eventual ventriculostomy. This approach was maintained due to suboptimal CSF characteristics, which prevented the successful placement of an internalized shunt following a second episode of ventriculitis. As a result, intrathecal antibiotic therapy was initiated.

**Table 2:** Summary of procedures and outcomes VPS: ventriculoperitoneal shunt; CSF: cerebrospinal fluid; EVS: external ventricular shunt; VS: ventricular shunt; VAS: ventriculoatrial shunt

Procedure No.	Description
1	Right VPS placement + central venous catheter placement
2	VPS revision + closure of subgaleal CSF fistula
3	VPS revision
4	VPS removal + parietal craniotomy + EVS placement
5	VPS placement (later replaced with EVS)
6	First device-associated ventriculitis
7	Resolution of ventriculitis
8	EVS removal + EVS placement
9	VS removal + right VPS placement
10	VPS revision + subgaleal puncture and drainage
11	VPS removal due to obstruction + EVS placement
12	Left femoral venous catheter placement
13	Craniotomy + EVS exchange + left frontal EVS placement
14	Left subclavian central venous catheter placement
15	EVS ventriculostomy exchange
16	Second device-associated ventriculitis + intrathecal antibiotic therapy
17	Resolution of ventriculitis
18	EVS removal + EVS replacement
19	VAS placement + right central venous catheter placement

**Figure 4:**
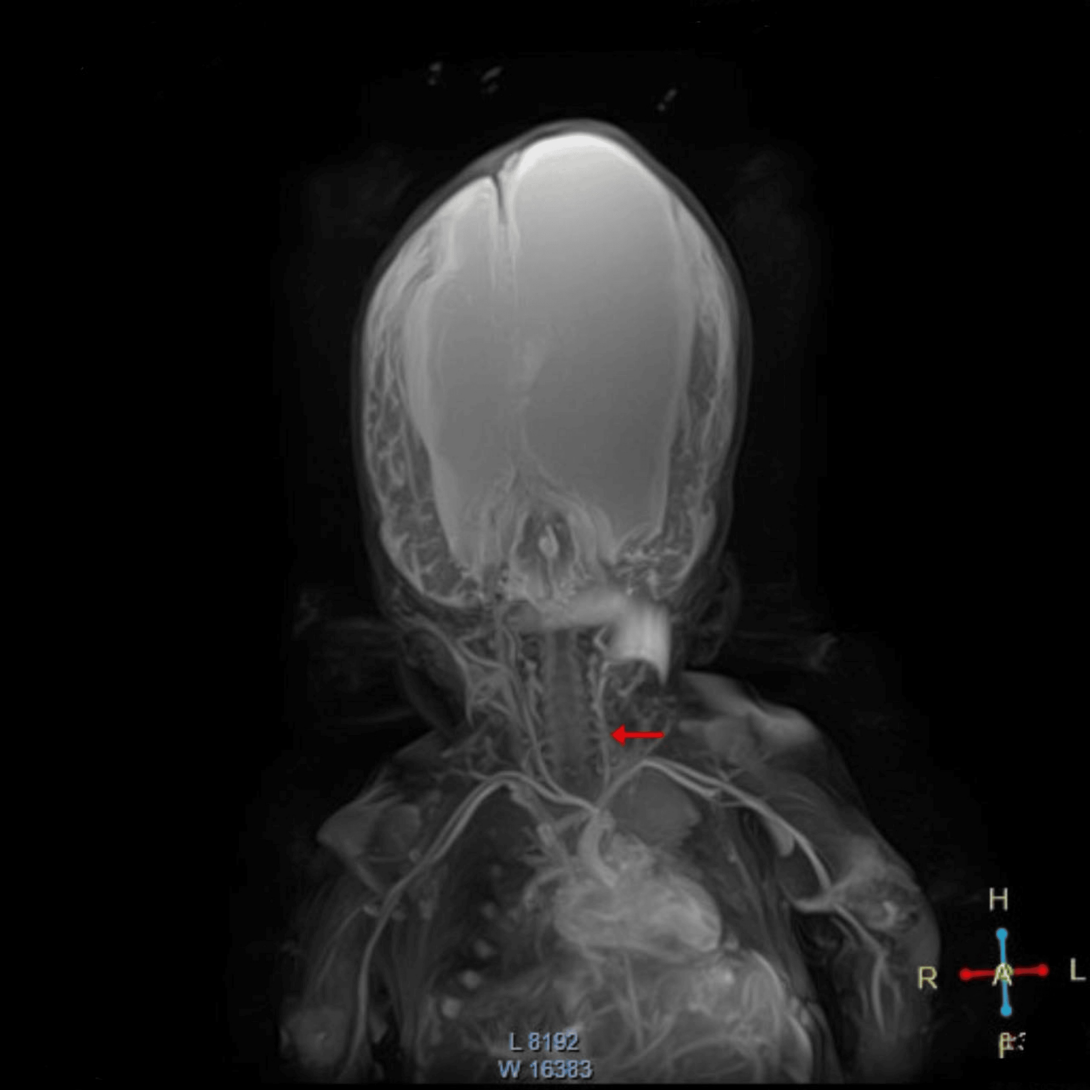
Cranial and cervical magnetic resonance angiography The red arrow indicates the left internal jugular vein, showing a reduction in caliber in the lower and middle thirds of the neck, suggesting obstruction, with recovery of caliber in the upper third of the neck, just before the ipsilateral jugular foramen.

Given the patient’s history of multiple interventions, recurrent infections involving various drainage systems, and the peritoneum’s limited capacity to absorb CSF, a multidisciplinary team recommended the placement of a fluoroscopy-guided VAS. To assess vascular suitability, a computed tomography angiography (CTA) of the neck and brain was performed (Video 1 and Figure 4), which revealed mild stenosis of the left IJV. This finding was attributed to prior use of the vessel for central venous catheterization, as documented in Table 2, and it guided the selection of the cannulation site for the procedure.

**Video 1:** Preoperative MRA of the skull and neck MRA: magnetic resonance angiography

After three consecutive CSF samples were confirmed sterile, the patient was taken to the operating room for VAS placement.

Description of surgical technique

During the preoperative phase in the neurosurgical planning laboratory, the surgical team attempted to insert the distal catheter of a Medtronic Medium-Pressure Shunt using 6 Fr and 7 Fr brachial introducers. However, the catheter diameter exceeded the capacity of these introducers. While an 8 Fr brachial introducer would have been suitable, it was unavailable. An alternative was selected in the form of an 8 Fr femoral introducer (2.67 mm diameter, 11 cm length, Lepu Medical, China). To adapt it for use in the upper extremity, the distal portion was trimmed to approximately 2 cm, allowing smooth passage of the guide wire and maintaining vascular access. This adjustment proved to be a practical, low-cost solution, and the introducer was aligned with the vascular path to ensure proper catheter placement.

**Figure 5:**
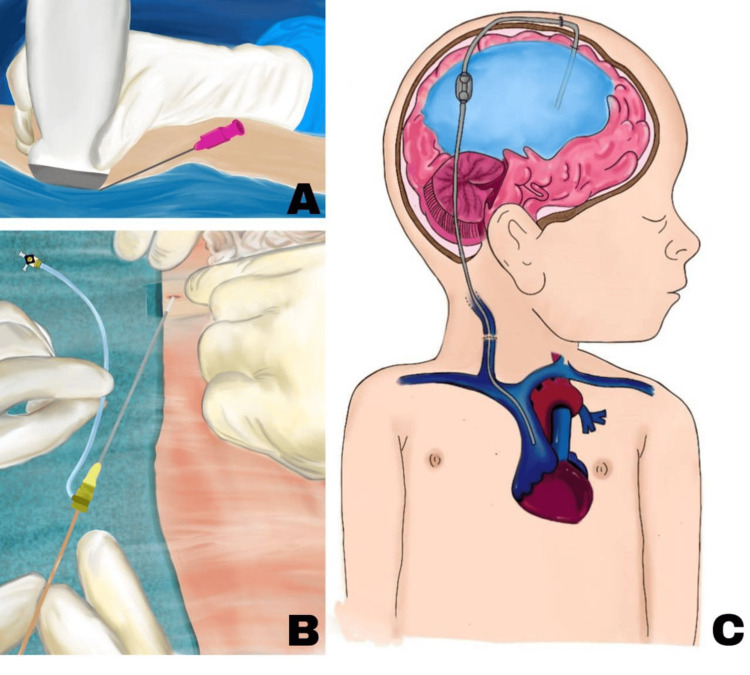
Artistic illustration demonstrating the key stages of ventriculoatrial shunt placement A: Ultrasound-guided puncture of the left internal jugular vein. B: A close-up view of the 8 Fr femoral catheter insertion using the modified Seldinger technique. The guidewire is advanced through the vascular introducer, illustrating the careful manipulation and alignment required for accurate placement. C: A lateral view of a pediatric patient showing the complete ventriculoatrial shunt system. The ventricular catheter is positioned within the lateral ventricle, connecting to a subgaleal reservoir. The distal catheter is tunneled subcutaneously and terminates at the right atriocaval junction, accurately placed within the venous system. Credit: Image created by the author Emmely Alexandra Prado

In the operating room, the patient was positioned supine with the head turned to the right. After induction of general anesthesia, aseptic preparation was performed on the cranial region and the left side of the neck. Using intraoperative ultrasound (Figure 5A), the left jugular vein was successfully punctured (Figure 5B), with venous access confirmed through visible blood return. This technique reduced access time compared to conventional approaches.

**Figure 6:**
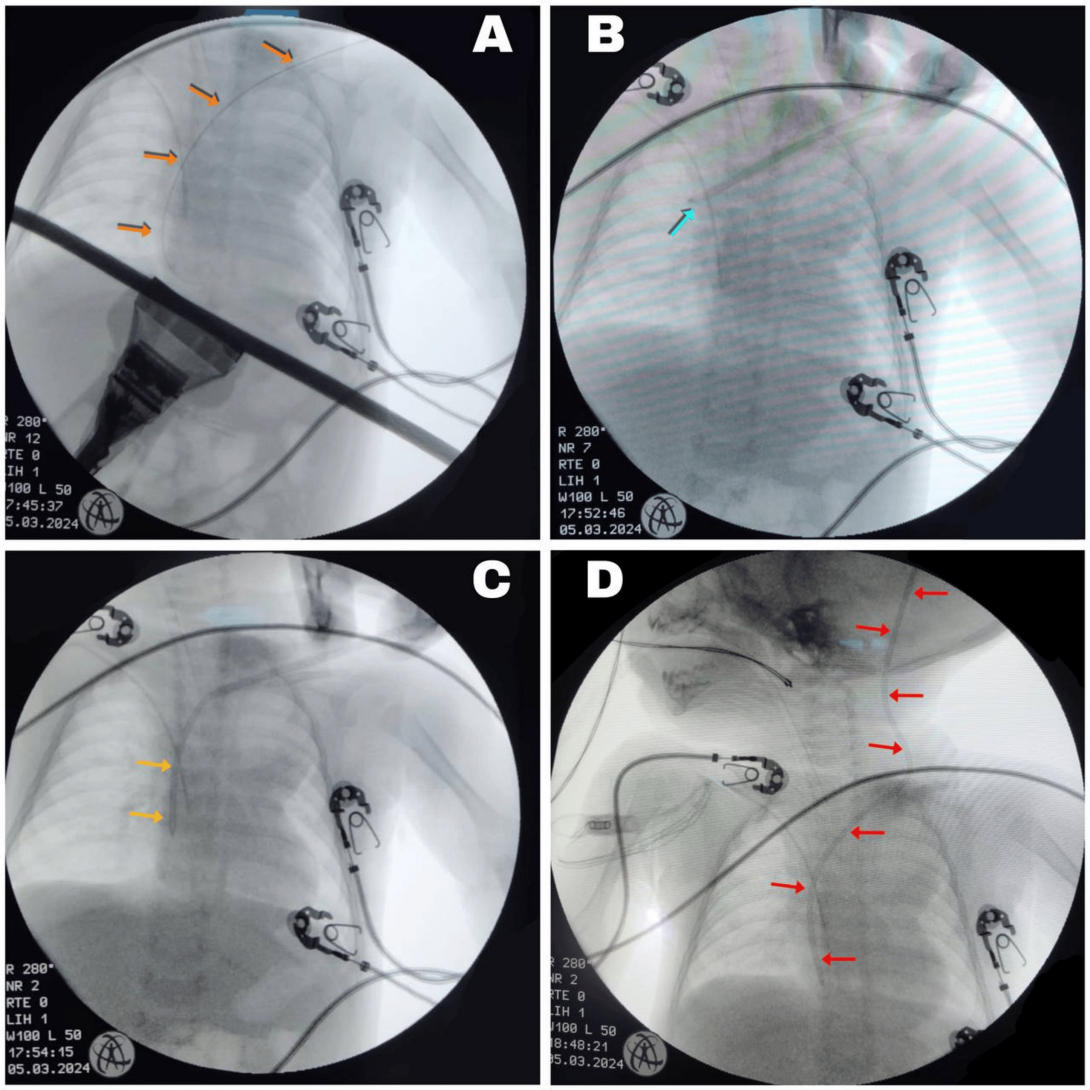
Fluoroscopic images during atrioventricular shunting A: Metal guide at the level of the right atrium (orange arrows). B: Tip of the distal catheter positioned in the right atrium, indicated by the tip of the turquoise arrow. C: Distal catheter tunneled in the right atrium, with its path marked by yellow arrows. D: Reservoir connected to the distal catheter, with the path of the ventriculoatrial shunt indicated by red arrows.

Intraoperative fluoroscopy was used to obtain anteroposterior (AP) and lateral projections, with the AP projection angled 5° craniocaudally. Three imaging modalities were employed: RoadMap, EchoNavigator, and standard fluoroscopy. Using the Seldinger technique, a guide wire was introduced (Figure 6A).

Following vascular access with the modified introducer previously discussed, AP fluoroscopy confirmed the optimal placement of the distal catheter at the right atriocaval junction (Figure 6B). During subsequent tunneling, the proximal catheter was guided cephalocaudally through the subgaleal space (Figure 6C). While this was performed, the distal catheter in the right atrium was continuously irrigated with 0.9% saline (1 mL/min using a 10 mL syringe) to maintain patency during cerebral ventricle cannulation. 

A horseshoe-shaped scalp incision was made over the left parietal bone, and a trephine was used to create a cranial opening. The dura mater was incised using electrocautery, and the cerebral ventricle was cannulated in a single attempt with the assistance of the Medtronic StealthStation S8 Neuronavigator (Medtronic, USA). High-pressure, clear CSF outflow confirmed accurate ventricular access. The ventricular catheter and reservoir were then positioned and secured with 4-0 Prolene sutures, followed by the connection and fixation of the distal catheter, as depicted in the artistic illustration shown in Figure 5C. Final fluoroscopic imaging confirmed the appropriate placement of all components (Figure 6D). 

The EVS system from the right frontal region was removed, and the catheter insertion site was sutured. The surgical field was irrigated, closed in layers, and sterile dressings were applied. The right central venous catheter was replaced without complications. 

Postoperative course

**Figure 7:**
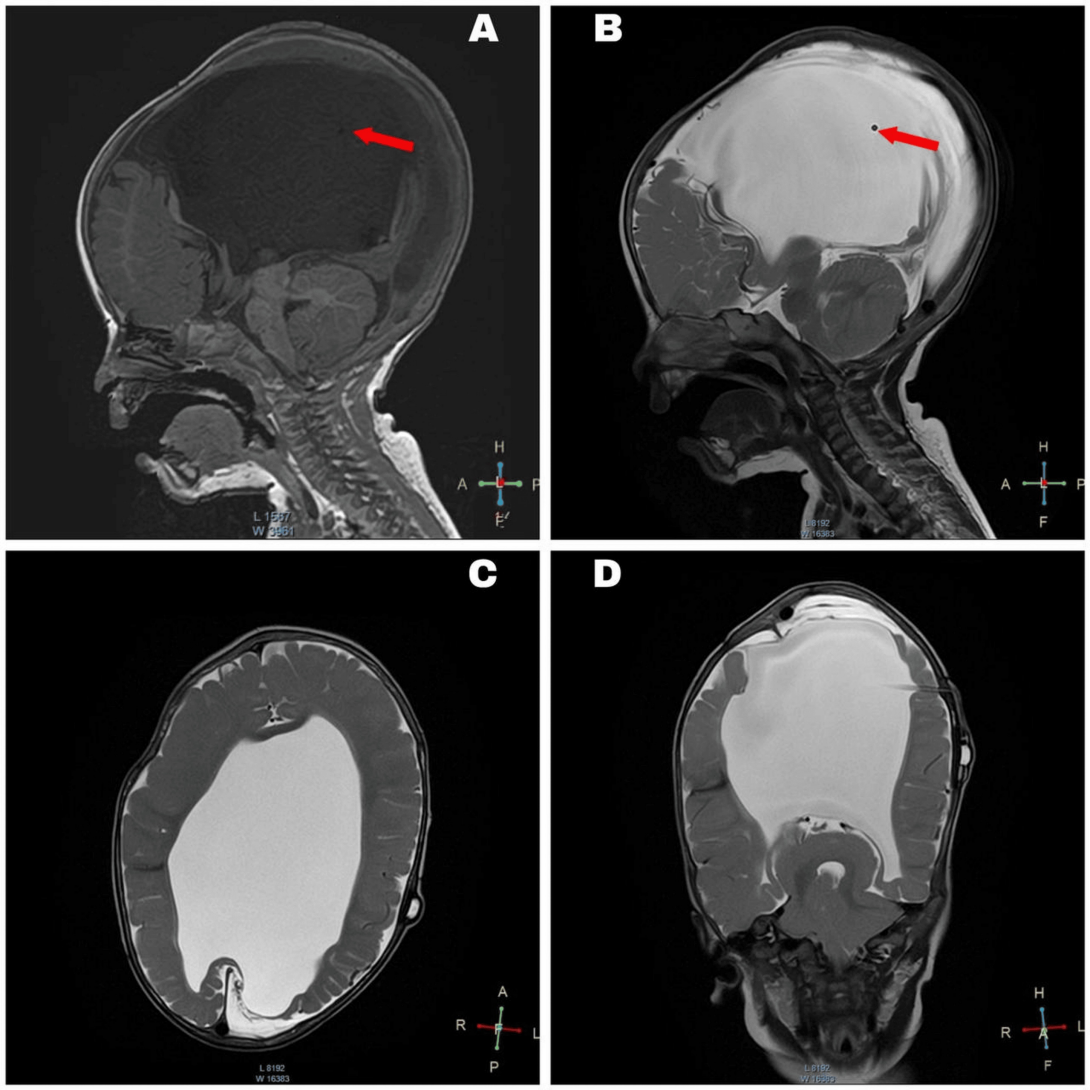
Follow-up magnetic resonance imaging of the brain at 12 months A-B: Sagittal view; red arrows indicate ventriculoatrial shunt catheter placement. C: Axial view. D: Coronal view; severe and asymmetric supratentorial hydrocephalus.

The patient demonstrated satisfactory recovery postoperatively. After a 153-day hospital stay, the patient was discharged. A multidisciplinary follow-up was scheduled, including visits with pediatrics, pediatric cardiology, and neurosurgery every two months for six months. Weekly physical therapy was initiated, and the patient showed significant improvement in head control, turning, and independent movement. At 12 months of age, the patient weighed 8.1 kg, measured 77 cm in length, and had a head circumference of 49.5 cm. No complications were reported during follow-up, and the latest clinical-radiological evaluation confirmed the proper function of the VAS (Figure 7).

## Discussion

VPSs remain the gold standard for treating congenital hydrocephalus; however, their long-term reliability is particularly compromised in neonates, where peritoneal immaturity often leads to early dysfunction [7]. In this case, the initial failure of the VPS was attributed to such limitations. Additionally, HPE, a known contributor to CSF hyperproduction due to increased choroid plexus activity, may have exacerbated system overload and failure [3,10]. In neonates with these conditions, VPS dysfunction rates can reach 40-50% within the first two years, with ventriculitis incidence exceeding 8% [11,12]. These challenges significantly contributed to the patient’s prolonged hospital stay of 153 days [11].

Recent comparative studies show that complication and revision rates are exceptionally high in pediatric shunt procedures, particularly with alternative distal sites such as VPS and VAS [2]. This underscores the necessity of considering alternative therapeutic approaches, including endoscopic third ventriculostomy (ETV) with or without choroid plexus coagulation (CPC), which has re-emerged as an option in select cases due to its minimally invasive nature [3]. This disparity further emphasizes the need for alternative treatments, such as ETV and CPC. Although promising, these techniques were contraindicated here due to recurrent ventriculitis and multiple early shunt failures, both known to increase their failure risk [13,14].

VASs become a rational alternative when both ventriculoperitoneal and ETV options are contraindicated. A recent meta-analysis involving 4,304 predominantly pediatric patients revealed no significant difference in mortality or revision rates between VAS and VPS and highlighted the superior resistance of VASs to dysfunction and obstruction - two key predictors of long-term success [15]. Hemodynamically, VASs are particularly well-suited for neonates, given that right atrial pressure (3-5 mmHg) is comparable to or lower than intra-abdominal pressure (4-10 mmHg) [16,17], offering a favorable pressure gradient for CSF drainage.

In addition to the previously discussed aspects, when examining general complication rates in pediatric patients, such as infection and shunt-related mortality, current evidence does not reveal statistically significant differences between VAS and VPS. The meta-analysis by Oliveira et al., involving a predominantly pediatric cohort, reported comparable infection rates (RR = 0.67; 95% CI: 0.36-1.25; I² = 74%) and mortality rates (RR = 2.11; 95% CI: 0.68-6.60; I² = 56%) between both shunt systems [15]. This discrepancy suggests the need for careful patient selection and meticulous surgical technique, which can make VASs a safe and effective alternative, highlighting the importance of a stringent, multidisciplinary approach.

Following the risk-benefit assessment mentioned previously, it is essential to address the specific cardiopulmonary compliance regarding VASs. These complications can be mitigated through meticulous surgical techniques, as employed in the present case. The most common ones include catheter malposition, thrombosis, migration of the catheter, and arrhythmias, which are often related to the proximity of cardiac structures [18].

Compared to traditional open venous access, this percutaneous approach is associated with a markedly shorter operative time, up to 64% less in some reports [19], and a lower incidence of infections, thrombosis, and embolic events [19]. The technique also improves cosmetic outcomes and simplifies future revisions by avoiding scar tissue dissection. When paired with ultrasound guidance, success rates improve even further, reaching up to 85%, and the likelihood of arterial puncture, pneumothorax, or hemothorax is significantly reduced [20,21].

The technique used in this case was inspired by adaptations described by Thirumal et al., who detailed the utility of a modified Seldinger technique for VAS insertion when conventional tools were insufficient [22]. To accommodate anatomical limitations, we employed a trimmed 8 Fr femoral introducer as an access sheath - a practical, cost-effective solution that enabled atraumatic cannulation of the left IJV. Fluoroscopy played a critical role in confirming the real-time positioning of the distal catheter at the atriocaval junction, thereby reducing the risk of malposition and optimizing long-term patency [19-20].

Ultimately, this case highlights that with judicious case selection, adherence to updated procedural protocols, and a collaborative multidisciplinary approach, VASs can provide a viable and safe alternative in complex neonatal hydrocephalus cases where traditional options are exhausted or contraindicated.

## Conclusions

While the VPS remains the gold standard for managing hydrocephalus, the VAS represents a critical alternative in cases of peritoneal insufficiency or recurrent abdominal complications. In this context, the integration of intraoperative fluoroscopy and ultrasonographic guidance - combined with the adaptation of a modified catheter system - has significantly improved the precision, safety, and feasibility of VAS placement. This minimally invasive technique enables accurate positioning of the distal catheter at the atriocaval junction, minimizes operative time and associated costs, ensures effective hemostasis through brief compression, and provides reliable vascular access with reduced risk to adjacent structures. These advancements highlight the importance of imaging-guided, anatomy-specific strategies in hydrocephalus management, particularly for complex or high-risk pediatric patients.
